# Near-Complete Airway Obstruction Caused by a Retropharyngeal Hematoma Following Anterior Cervical Discectomy and Fusion Managed With Nasotracheal Intubation

**DOI:** 10.7759/cureus.108546

**Published:** 2026-05-09

**Authors:** Yui Somura, Takehito Sato

**Affiliations:** 1 Department of Anesthesiology, Nagoya University Hospital, Nagoya, JPN

**Keywords:** anesthesiology, anterior cervical discectomy and fusion (acdf), anterior cervical spine surgery, awake fiberoptic nasal intubation, hematoma, nasotracheal intubation, near-complete airway obstruction, retropharyngeal hematoma

## Abstract

Postoperative retropharyngeal hematoma after anterior cervical spine surgery is a rare but potentially life-threatening complication because it can rapidly cause airway obstruction. In addition, it may result in an unanticipated difficult airway due to anatomical distortion.

A patient underwent an anterior cervical discectomy and fusion (ACDF) at the C3-C5 level for dropped head syndrome. Approximately one hour after surgery, the patient developed sudden dyspnea and agitation. Emergency reintubation was attempted; however, direct oral intubation was extremely difficult because the glottis had shifted superiorly due to severe pharyngeal compression. Fiberoptic nasotracheal intubation using a 6.5-mm endotracheal tube was successfully performed. Computed tomography (CT) revealed a large retropharyngeal hematoma causing near-complete airway obstruction. Postoperative retropharyngeal hematoma can cause sudden airway obstruction and an unanticipated difficult airway. Prompt recognition and appropriate airway management are essential.

## Introduction

Anterior cervical spine surgery is widely performed for cervical spine disorders and generally considered safe; however, various complications, including airway compromise, dysphagia, and nerve injury, have been reported [1,2]. Postoperative airway compromise remains one of the most serious complications, with a reported incidence ranging from approximately 0.2% to 6.1% [1-4].

Airway compromise after anterior cervical spine surgery may result from several mechanisms, including retropharyngeal hematoma, pharyngolaryngeal edema, graft displacement, and nerve injury [2,4]. Among these, retropharyngeal hematoma is particularly critical because it can rapidly compress the airway due to the limited anatomical space in the retropharyngeal region [1]. In addition to causing airway obstruction, retropharyngeal hematoma may distort airway anatomy and result in a difficult airway; however, this mechanism has not been sufficiently emphasized.

Herein, we report a case of acute airway obstruction caused by a postoperative hematoma following anterior cervical spine surgery, successfully managed with fiberoptic nasotracheal intubation.

## Case presentation

The patient was a 75-year-old woman, 140 cm tall and weighing 53 kg. She had a history of hypertension (currently receiving oral medication) and underwent an anterior cervical discectomy and fusion (ACDF) at the C3-C5 level for dropped head syndrome. The surgical procedure was uneventful, and the patient was extubated after surgery.

Approximately one hour postoperatively, the patient developed sudden dyspnea and agitation. Oxygen saturation decreased, and acute airway obstruction was suspected.

An intensivist attempted endotracheal intubation; however, direct oral intubation was extremely difficult because the glottis had shifted superiorly due to severe pharyngeal compression. Visualization of the glottis was markedly impaired. Fiberoptic nasotracheal intubation using a 6.5-mm endotracheal tube was successfully performed, and the airway was secured.

**Figure 1:**
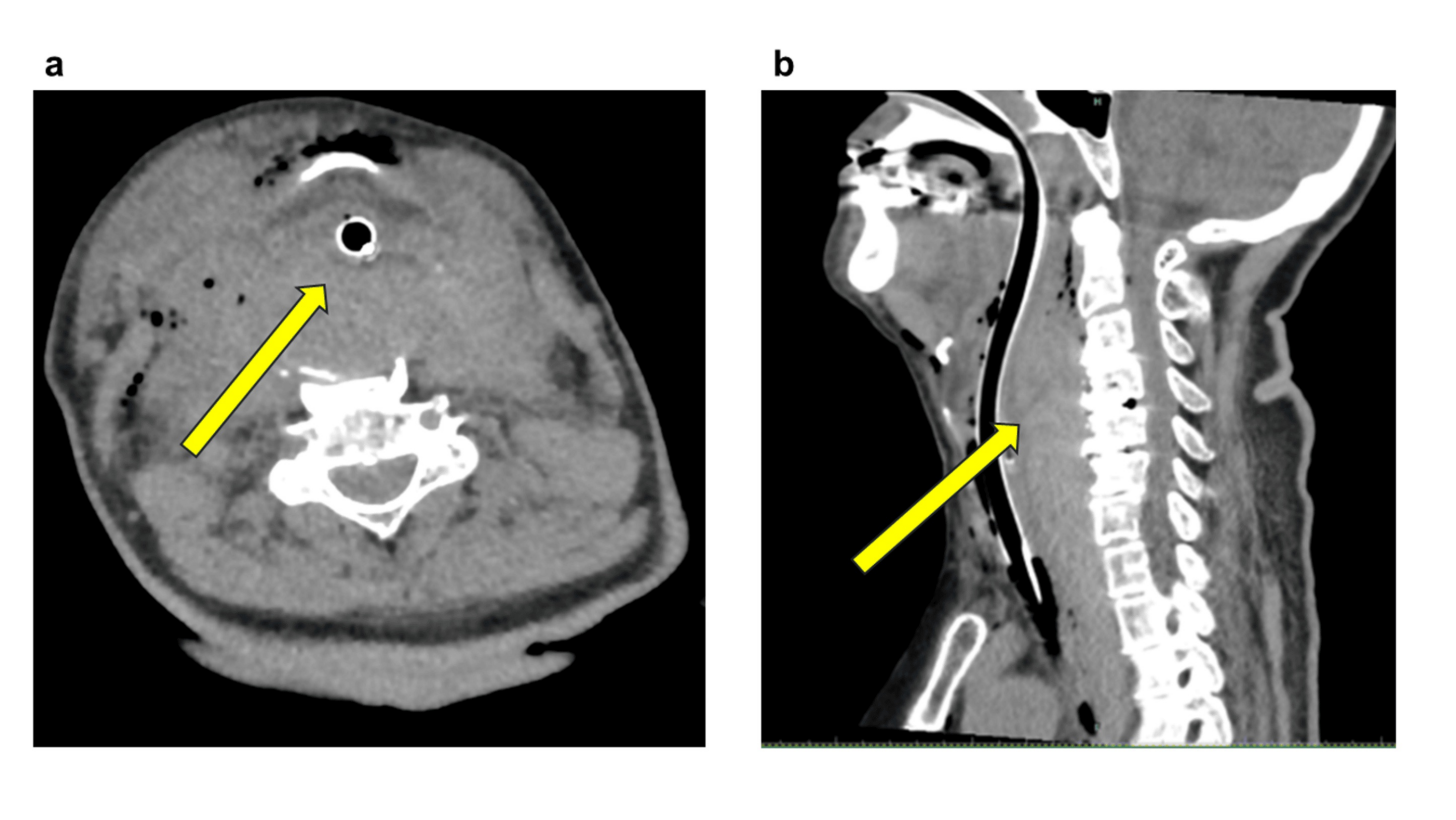
Computed tomography (CT) after emergency intubation CT images demonstrate a large retropharyngeal hematoma compressing the upper airway following anterior cervical discectomy and fusion (arrow). (a) Axial CT; (b) Sagittal CT

Computed tomography (CT) revealed a large retropharyngeal hematoma extending along the anterior surface of the cervical vertebral bodies. The hematoma caused marked compression of the upper airway, leaving only minimal airway space surrounding the endotracheal tube (Figure 1).

**Figure 2:**
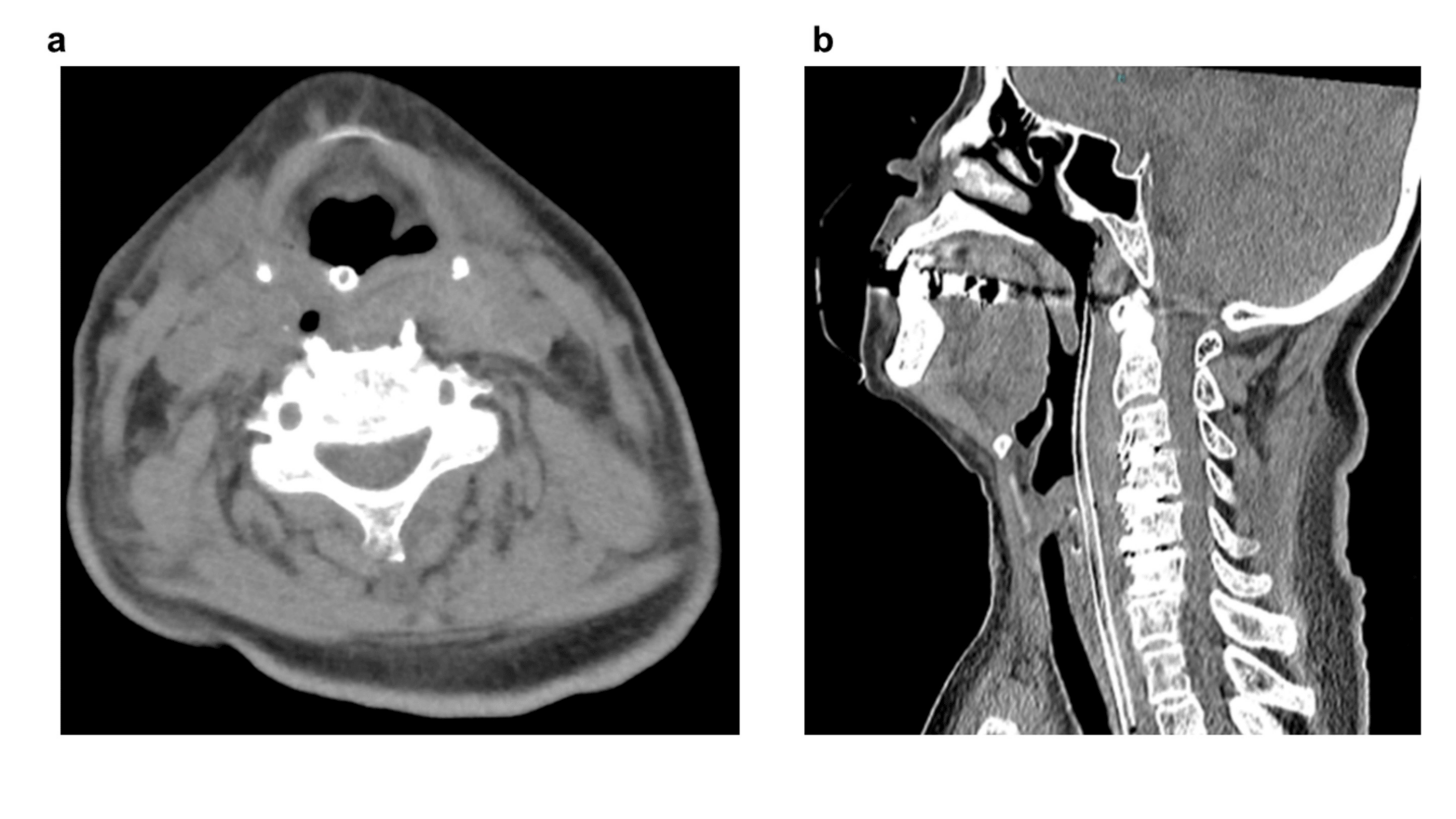
Follow-up computed tomography (CT) two days after surgery demonstrates complete resolution of the retropharyngeal hematoma with restoration of the airway. (A) Axial CT; (B) Sagittal CT

An emergency operation was performed immediately. Although no definite bleeding was identified, oozing was noted from the anterior surface of the C3 vertebral body, suggesting a bony origin. And the hematoma was evacuated. After decompression, she was readmitted to the ICU while intubated. A CT scan obtained the day after hematoma evacuation showed no soft tissue swelling, and bronchoscopy revealed no pharyngeal or glottic edema (Figure 2).

She was extubated two days after the reoperation. After extubation, no neurological sequelae, such as cognitive impairment or paralysis, were observed. The patient was discharged from the ICU on postoperative day 4 and from the hospital on postoperative day 10.

## Discussion

Postoperative airway compromise after anterior cervical spine surgery is uncommon but potentially life-threatening. Previous studies have reported an incidence ranging from approximately 0.2% to 6.1% [1-4]. Retropharyngeal hematoma is one of the most critical causes of early airway obstruction because it can rapidly compress the airway [1,2].

The retropharyngeal space is anatomically narrow, and even a small volume of bleeding may result in significant airway narrowing and distortion [1,2]. In addition to airway compression, hematoma formation may lead to tracheal deviation and displacement of the glottis, resulting in a difficult airway.

The timing of symptom onset is an important diagnostic clue. Airway compromise due to hematoma typically occurs within the first six to 24 hours after surgery [2,4]. In the present case, symptoms developed approximately one hour postoperatively, strongly suggesting acute hematoma formation.

A notable feature of this case was the superior displacement of the glottis caused by pharyngeal compression. This anatomical change significantly impaired visualization during direct laryngoscopy and resulted in a difficult airway. Although airway distortion has been described in previous reports, it has not been emphasized as a key mechanism [4,5].

Airway management in such cases can be particularly challenging. Direct laryngoscopy may fail due to anatomical distortion, and alternative airway techniques such as fiberoptic intubation, video laryngoscopy, or surgical airway may be required [6]. In this case, fiberoptic nasotracheal intubation was successfully performed.

Fiberoptic intubation is considered an effective technique for managing difficult airways in patients with cervical spine pathology or postoperative airway compromise [4]. Previous reports have described the successful use of fiberoptic techniques for airway rescue in cases of airway obstruction caused by postoperative cervical hematoma [7-9]. However, most reports do not specifically focus on the route of intubation, and the role of nasotracheal intubation in this setting has not been well discussed.

In the present case, nasotracheal fiberoptic intubation was successfully performed despite severe airway narrowing and anatomical distortion. Compared with oral intubation, the nasotracheal route may offer certain advantages in this context. First, the nasotracheal approach may provide a more favorable angle for visualization of the glottis when the airway is displaced anteriorly or superiorly. Second, the fiberoptic bronchoscope can be advanced more smoothly along the anatomical curvature of the nasopharynx, which may facilitate intubation under conditions of restricted oral access or distorted airway anatomy.

A notable feature of this case was the superior displacement of the glottis, which significantly impaired visualization during direct laryngoscopy. This mechanism has been described in previous studies as a consequence of pharyngeal compression and airway distortion [2,4], but has not been emphasized as a major contributor to difficult airway management. The present case suggests that clinicians should anticipate not only airway obstruction but also an unanticipated difficult airway due to glottic displacement in patients with postoperative cervical hematoma.

Although fiberoptic intubation is recognized as a valuable technique in difficult airway management, reports specifically describing the successful use of nasotracheal fiberoptic intubation in this clinical scenario remain limited. Therefore, this case highlights the potential usefulness of the nasotracheal fiberoptic approach as a rescue technique for securing the airway in patients with severe airway compromise after anterior cervical spine surgery.

Several risk factors for postoperative airway compromise have been identified, including advanced age, male sex, chronic pulmonary disease, multilevel surgery, and prolonged operative time [3,10]. Careful postoperative monitoring is therefore essential.

This case highlights that postoperative retropharyngeal hematoma should be recognized not only as a cause of airway obstruction but also as a cause of an unanticipated difficult airway.

## Conclusions

Postoperative retropharyngeal hematoma after anterior cervical spine surgery can cause sudden airway obstruction and a difficult airway due to anatomical distortion. In this case, transnasal fiberoptic intubation was successfully performed in the ICU. CT revealed marked upper airway obstruction due to the hematoma, with posterior displacement of the glottis, suggesting that conventional orotracheal intubation would have been difficult.

When asphyxiation due to postoperative hematoma is suspected, prompt airway management, including transnasal fiberoptic intubation, should be considered, followed by emergent surgical evacuation when indicated. In conclusion, clinicians should anticipate airway difficulty and prepare appropriate airway management strategies.
